# Quantifying Transmission Investment in Malaria Parasites

**DOI:** 10.1371/journal.pcbi.1004718

**Published:** 2016-02-18

**Authors:** Megan A. Greischar, Nicole Mideo, Andrew F. Read, Ottar N. Bjørnstad

**Affiliations:** 1 Department of Entomology and the Center For Infectious Disease Dynamics, The Pennsylvania State University, University Park, Pennsylvania, United States of America; 2 Department of Ecology & Evolutionary Biology, University of Toronto, Toronto, Ontario, Canada; 3 Department of Biology, The Pennsylvania State University, University Park, Pennsylvania, United States of America; 4 Fogarty International Center, National Institutes of Health, Bethesda, Maryland, United States of America; Emory University, UNITED STATES

## Abstract

Many microparasites infect new hosts with specialized life stages, requiring a subset of the parasite population to forgo proliferation and develop into transmission forms. Transmission stage production influences infectivity, host exploitation, and the impact of medical interventions like drug treatment. Predicting how parasites will respond to public health efforts on both epidemiological and evolutionary timescales requires understanding transmission strategies. These strategies can rarely be observed directly and must typically be inferred from infection dynamics. Using malaria as a case study, we test previously described methods for inferring transmission stage investment against simulated data generated with a model of within-host infection dynamics, where the true transmission investment is known. We show that existing methods are inadequate and potentially very misleading. The key difficulty lies in separating transmission stages produced by different generations of parasites. We develop a new approach that performs much better on simulated data. Applying this approach to real data from mice infected with a single *Plasmodium chabaudi* strain, we estimate that transmission investment varies from zero to 20%, with evidence for variable investment over time in some hosts, but not others. These patterns suggest that, even in experimental infections where host genetics and other environmental factors are controlled, parasites may exhibit remarkably different patterns of transmission investment.

## Author Summary

Malaria parasites are carried from host to host by blood-feeding insects, a process that requires some portion of the parasite population to develop into transmission forms that cannot replicate within the current host. The fraction of parasites specialized for transmission instead of replication (transmission investment) could change with each cycle of replication in response to changing conditions within the host. Measuring how transmission investment changes through time could help us understand how malaria spreads so efficiently through populations of human and other animals. However, transmission investment is usually impossible to measure directly and instead has to be estimated by comparing the number of transmission forms with total parasite numbers in blood samples. Here we use a model to simulate data from an infection—so that the true level of transmission investment is known—and test published methods for estimation. We find that existing methods do not accurately estimate transmission investment from simulated data, and we propose a new statistical method that works substantially better. When applied to rodent malaria data, our method suggests that transmission investment can vary substantially over the course of infection, with notably different patterns of allocation across hosts.

## Introduction

Parasite life cycles involve both proliferation within-hosts and transmission to new hosts. Many microparasites have evolved specialized transmission forms—including protozoa, fungi and viruses—giving rise to a tradeoff between proliferation within the host and onward transmission [1]. Since transmission stage production comes at the cost of within-host replication, it represents a fundamental aspect of parasite fitness and a potential target for disease intervention efforts, provided the proximate cues and evolutionary drivers of allocation patterns can be identified (reviewed in [2]). Information is needed on the range of strategies parasites can employ, what cues in the within-host environment (if any) trigger changes in allocation, and how quickly the parasite population can respond to perturbations, such as drug treatment of the host. None of this is attainable without robust methods to estimate transmission investment from time series data. Here we use simulated data—where the true pattern of transmission investment is known—to show that current methods for estimating allocation [3–5] can be seriously misleading, inferring complicated strategies where none exist. We therefore develop a better inferential method by expanding recent regression methods [6] and apply this method to real data, revealing unexpected diversity in the transmission investment strategies of malaria parasites in a highly-controlled setting of rodent malaria infections.

Malaria parasites (*Plasmodium* species) replicate within red blood cells of their vertebrate host, developing into mature stages called schizonts that burst to release merozoites capable of invading other red blood cells [7]. *In vitro* assays of the human malaria parasite *P. falciparum* suggest that all of the merozoites emerging from a given schizont will be committed either to the transmission route—invading a red blood cell and developing into a sexual gametocyte that can be passed onto the vector in a blood meal—or to further in-host proliferation by invading a red blood cell, maturing into another schizont and subsequently bursting to release more merozoites [8]. Gametocytes are specialized for sexual reproduction in the midgut of the vector and cannot infect red blood cells [9], so that investment in transmission should be costly to within-host replication [10, 11]. Mature gametocytes can be readily distinguished from asexual forms by molecular methods (e.g., [12, 13]) or microscopy (e.g., [8]).

**Fig 1:**
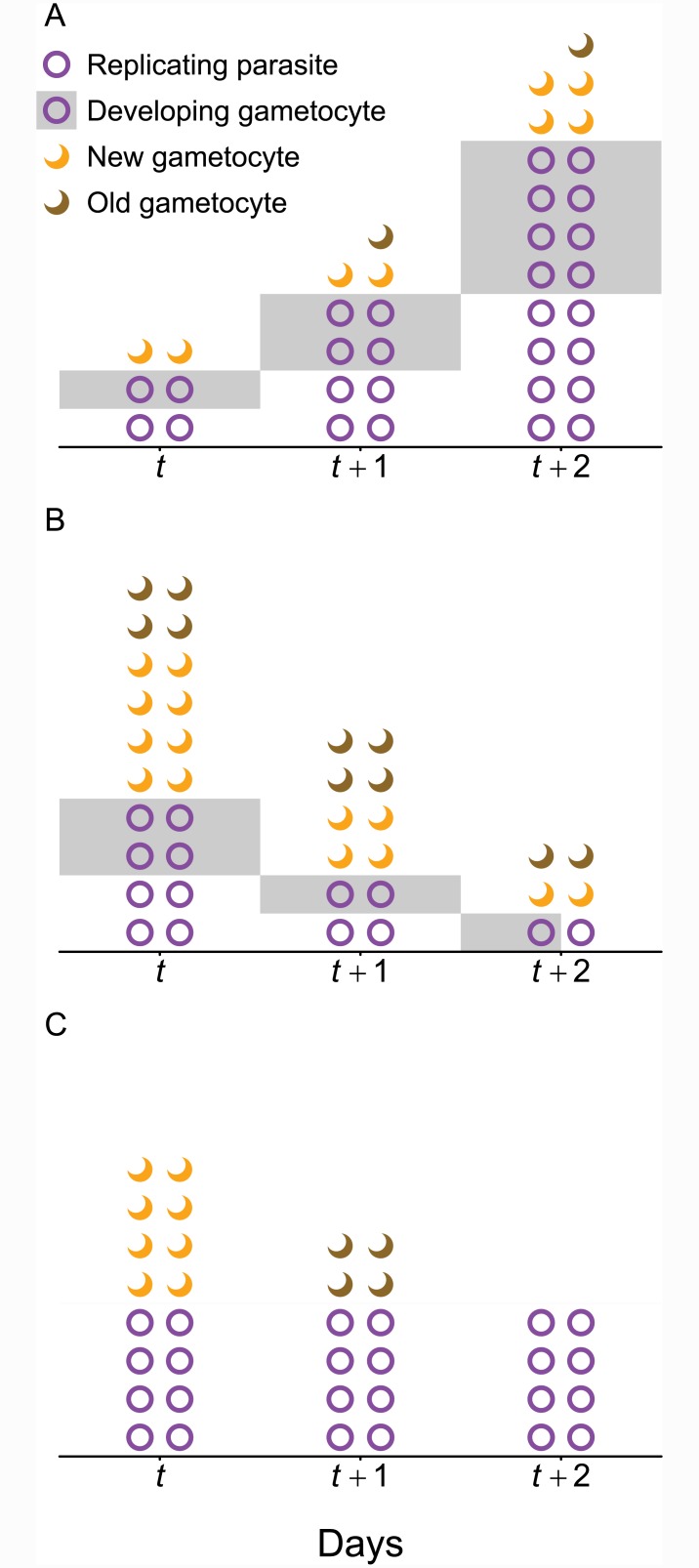
Gametocyte numbers can increase (A) or decrease (B, C) even when transmission investment is constant over the time period observed. For clarity, we assume that replication and gametocyte development both require one day and 50% of new gametocytes persist for an additional day (“Old gametocytes”). (A) Gametocyte numbers may increase because the parasite population is expanding, even though the percentage of each cohort committed to transmission remains 50%. That is, parasites in half of the newly invaded red blood cells (circles) will develop into gametocytes (gray shading) while in the other half (unshaded), parasites will replicate and each generate four newly invaded red blood cells the following day. (B) In contrast, if the parasite population is declining so that only half the parasites can replace themselves (for example, because red blood cells have been depleted), gametocyte numbers can decrease while the transmission investment remains constant at 50%. (C) Alternately, the gametocytes observed on a given day (e.g., *t*, *t* + 1) may have been produced by previous cohorts with no ongoing transmission investment (and hence no developing gametocytes) during the time period observed. In this case, gametocyte numbers are declining due to mortality rather than any change in transmission investment during the observation window. We use circles to indicate both replicating parasites and developing gametocytes since those forms are often indistinguishable in *P. chabaudi*; likewise, crescents indicate both newly-matured and ‘old’ gametocytes generated by previous cohorts since those cannot be differentiated in either *P. chabaudi* or *P. falciparum*.

Transmission investment is defined as the fraction of a given cohort of parasites that commit to differentiation into gametocytes [14], a proportion known as the “conversion rate” by convention [8]. Conversion rates can be measured directly *in vitro* by fixing cells in a monolayer and observing their development [8] or by using molecular markers to detect gametocyte production from a single cohort of parasites [15]. Critically, these methods are only able to assess transmission investment for a single cohort of parasites and only in the highly-controlled environment of *in vitro* culture. Characterizing changes in allocation over the course of infection requires time series data, but gametocyte dynamics are driven by parasite proliferation and gametocyte longevity in addition to transmission investment. We use a heuristic model to illustrate that gametocyte numbers may increase or decrease while transmission investment holds steady (Fig 1). Even if transmission investment is consistently 50%, gametocytes may only rarely compose 50% of the parasite population (Fig 1A and 1B). Thus, while it is tempting to draw inferences from relative numbers of gametocytes and asexual stages from a single point in time (e.g., [16]), the presence of gametocytes only confirms that some transmission investment occurred previously and cannot be used to gauge the level of transmission investment or how allocation has changed over time.

Accurately estimating transmission investment requires linking gametocytes with their progenitor cohort. Many, but not all, malaria species exhibit discrete cohorts of schizonts, which develop synchronously and burst in unison to generate another cohort of infected red blood cells (reviewed in [17, 18]). Synchrony is helpful for quantifying transmission investment because asexual parasites can be separated into identifiable cohorts, but it is not possible to distinguish newly-matured gametocytes from those produced by previous cohorts (in contrast to the color-coding used for clarity in Fig 1). In addition to being well-synchronized [19], the problem of gametocyte carryover is likely to be minimized in the rodent malaria *P. chabaudi*, where the reported gametocyte half-life is 14 hours [20]. However, *P. falciparum* produces gametocytes that can circulate for more than six days (reviewed in [9]), much longer than the two days required for the asexual life cycle, meaning that gametocytes from several asexual cohorts are likely to be present simultaneously even in a highly synchronized infection. We focus on the comparatively simple case of *P. chabaudi* to show that even modest gametocyte carryover can severely bias estimates of transmission investment.

### Current methods for estimating transmission investment

PCR methods have been developed to quantify abundance of both asexual parasites and mature gametocytes in *P. chabaudi* infections [13, 21], and a variety of techniques have been developed to quantify transmission investment from these time series data. A recent study used linear mixed effects models to examine how transmission investment varied with red blood cell availability [22]. Other studies attempt to estimate transmission investment explicitly, because direct estimates are conceptually appealing and easily incorporated into modeling efforts (e.g., [18]). Such direct estimates infer transmission investment *c* from time series of gametocyte abundance and total parasite numbers, making use of the fact that infected red blood cells take two days to develop into mature gametocytes [23]. The simplest method that accounts for the time lag between transmission investment and gametocyte maturity would be

$$c_{t}=\frac{G_{t+2}}{A_{t}},$$ (1)

where *A*_*t*_ is the total number of red blood cells invaded, by either sexually- or asexually-committed merozoites at time *t*, and *c*_*t*_ is the fraction of invaded cells that develop into mature gametocytes two days later (*G*_*t*+2_). This simple estimate requires negligible mortality during the two day window of development. This method is similar to ones commonly used for *P. falciparum in vitro* (e.g., [4]), where early stage gametocytes can be identified and ignoring mortality is likely to be a fair approximation.

*In vivo*, neglecting mortality is thought to be too unrealistic an assumption, so methods attempting to correct for mortality have been proposed. Buckling *et al*. [3] derived a commonly used method (e.g., [5, 24, 25]), assuming that the number of gametocytes at time *t* + 2 can be calculated as

$$G_{t+2}=sc_{t}mA_{t}$$ (2)

where *m* is the burst size (i.e., the number of merozoites emerging from a burst red blood cell) and *s* is the proportion of parasites surviving development. Since the asexual cycle takes one day [26], two cycles of asexual growth occur during gametocyte maturation, so that the number of asexual stages at time *t* + 2 is

$$A_{t+2}=s^{2}{\left(1-c_{t}\right)}^{2}m^{2}A_{t}.$$ (3)

Buckling *et al*. [3] solve for transmission investment by combining Eqs 2 and 3:

$$c_{t}=\frac{\frac{G_{t+2}}{A_{t}}}{\sqrt{\frac{A_{t+2}}{A_{t}}}+\frac{G_{t+2}}{A_{t}}}$$ (4)

A subsequent review suggested that the appropriate time lag would be three days, or three cycles of asexual growth, since transmission investment occurs in the cycle prior to gametocyte development [2]. Thus the parasites that are committed at time *t* will burst out and invade new red blood cells before taking two days to develop into gametocytes, so that transmission investment should be defined as

$$c_{t}=\frac{\frac{G_{t+3}}{A_{t}}}{\sqrt[{3}]{\frac{A_{t+3}}{A_{t}}}+\frac{G_{t+3}}{A_{t}}}.$$ (5)

These methods for inferring transmission investment are expected to be sensitive to the assumption that both gametocytes and asexual stages are equally likely to survive development (i.e., the same *s* is used in both Eqs 2 and 3). This assumption would be violated by differential immune clearance [2, 3], which is a concern since immunity predominately targets asexual parasites (reviewed in [27]). The same issues would apply to an even greater degree in *P. falciparum*, where gametocytes take much longer to mature [28].

If differential mortality of gametocytes and asexual stages is a problem, it could be addressed by detecting gametocyte development earlier. While not yet detectable in *P. chabaudi*, early signals of gametocyte development can be detected in the human malaria *P. falciparum*[12, 16]. We simulate data assuming early detection of gametocyte development and find that it does not improve estimates of transmission investment except under highly-restrictive conditions (S1 Text, S1 and S2 Figs). Whether time series include mature or immature gametocytes, currently-described methods fail to account for the carryover of gametocytes produced by previous asexual cohorts. While this bias can be addressed by fitting a detailed mechanistic model to time series data (e.g., from neurosyphilis patients, [29]), we develop an alternative approach requiring fewer strict assumptions about the biology.

## Results & Discussion

### Failure of existing methods

We simulated dynamics in *P. chabaudi*-like infections of mice using a previously described model [18] that gives current methods the best possible chance of working by incorporating the key assumptions thought to yield reliable estimates of transmission investment. Specifically, we assumed a highly synchronized infection and, at least in initial simulations, no immune clearance. The model does, however, include homeostatic regulation of red blood cell abundance, as well as the capability to incorporate immune clearance of infected red blood cells. For the simulations, we assume that the duration of parasite development (both sexual and asexual) is fixed with no variation, so that a high degree of synchrony is maintained [18]. From high-resolution simulated data, we sampled daily counts of total parasite numbers and gametocyte abundance, assuming no sampling error.

**Fig 2:**
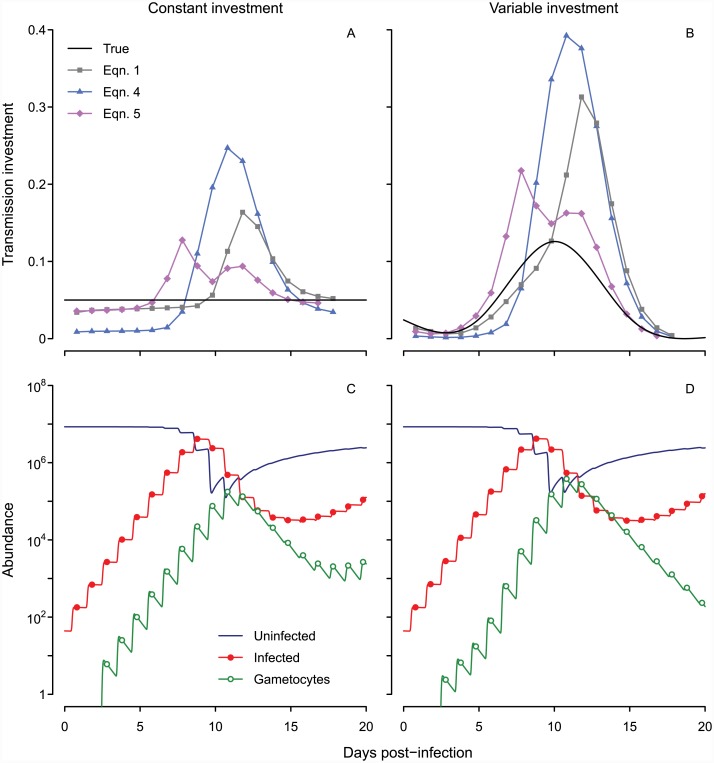
Current methods for inference yield spurious oscillations whether the true transmission investment is constant or variable. Estimates of transmission investment by different methods are shown when the actual level is fixed at 5% (solid black line, A) or variable (B). The corresponding dynamics of uninfected and infected red blood cells (dark blue line and red closed circles, respectively) and mature gametocytes (green open circles) are shown below (C, D). Infected red blood cell abundance includes asexual parasites and developing sexual forms, but not mature gametocytes. We assume a mean gametocyte lifespan of about 20 hours, equivalent to the experimentally-derived half-life of 14 hours for *P. chabaudi* gametocytes [20].

All three inference methods (Eqs 1, 4 and 5) return qualitatively incorrect patterns (Fig 2A and 2B) and cannot distinguish between constant and variable patterns of allocation. Even when the true level of transmission investment is fixed at 5% (in the range reported previously for *P. chabaudi*, [5]), the estimated value rises as parasite numbers increase, making it appear as though parasites are modulating their investment in response to changing environmental conditions. The spurious changes in estimated transmission investment are amplified when we simulate a variable pattern of investment (Fig 2B and 2D). Whether this investment pattern is plausible (and hence a good choice to test prescribed methods) cannot be evaluated, at least with these methods. The estimated values deviate so much from the true pattern that it is unclear which aspects (if any) of current expectations regarding transmission investment can be relied upon. The limitation typically thought to introduce error—differential mortality of asexual and sexual forms (e.g., [3])—does not apply here. In the simulated dynamics, developing sexual stages and asexual stages are subject to the same low background mortality rate, and mature gametocytes persist approximately 20 hours on average (equivalent to a the 14 hour half-life reported by [20]), similar to the 24 hour period required for infected red blood cells to burst. Instead our analysis suggests that the blurring together of synchronized cohorts creates bias. Simulated gametocytes peak each day (Fig 2C and 2D), but abundance does not drop to zero between peaks because gametocyte lifespans are exponentially-distributed. Thus, a mean lifespan of 20 hours equates to 30% of gametocytes persisting from one time point to the next. A major part of the problem lies in incorrectly attributing the observed gametocyte population to a single cohort, a complication emerging from the parasite life cycle (Fig 1). The magnitude of the error depends on the number of gametocytes produced previously; that is, the errors in gametocyte abundance are autocorrelated, a familiar problem in parasitology (reviewed in [30]).

These complexities call into question previous work quantifying transmission investment in malaria parasites, both *in vivo* and *in vitro*. A recent study found that transmission investment in *P. chabaudi* increased with declining red blood cell numbers, assuming independent residuals [22]. That approach is likely to generate spurious patterns because it does not address the problem of autocorrelated errors in gametocyte counts. Using our simulated data, we can generate the appearance of a negative correlation between red blood cell numbers and transmission investment (Fig 2)—even when transmission investment is constant—by failing to account for autocorrelation in gametocyte abundance. The error due to gametocyte carryover is likely to increase with time if there is ongoing gametocyte production, creating the appearance of increasing transmission investment as the infection progresses and within-host conditions deteriorate (i.e., terminal investment, reviewed in [2]). Conversion rates have been reported to increase late in infection *in vivo* (*P. chabaudi*, [3]), a pattern that could represent either an artifact of temporal autocorrelation or strategic allocation on the part of parasites. The problem of temporal autocorrelation is likely to be more pronounced in *P. falciparum*, given the long lifespan of gametocytes (reviewed in [9]). Assessing transmission investment by a single parasite cohort (e.g., by fixing parasites in a monolayer, [8]) circumvents this problem, but other approaches may be needed when more than one parasite cohort is considered. While cultured *P. falciparum* parasites appear to alter transmission investment when they are at risk of drug clearance (using an equation analogous to Eq 1, [4]), methods that account for temporal autocorrelation may reveal a different pattern.

Blurring of gametocyte cohorts may likewise complicate sex ratio estimates, particularly since male gametocytes persist twice as long as females [20]. Our simulations assumed a uniform mortality rate for all gametocytes, set to yield the mean lifespan of male and female gametocytes reported in [20], and found that sufficient gametocytes persisted long enough to bias the inferred transmission investment. Under more realistic assumptions, male gametocytes would be more likely (and female gametocytes less likely) to persist through multiple time points. Researchers have observed sex ratios less female-biased than expected from theory, and while adaptive explanations have been proposed (e.g., [31]), our results hint that part of the discrepancy may be explained by the longer lifespan of male gametocytes [20, 32].

### A new method

Since current methods are inaccurate, we develop an alternative approach by elaborating the recently proposed time series model of in-host malaria dynamics for *P. chabaudi*[6]. Asexual growth can be modeled via the effective propagation number, *P*_*e*, *t*_ for each cycle of asexual proliferation:

$$I_{t+1}=P_{e,t}I_{t}S_{t}$$ (6)

where *I*_*t*_ indicates the total number of infected red blood cells excluding any mature gametocytes, and *S*_*t*_ is the number of uninfected red blood cells. Thus *I*_*t*_ represents mainly asexual parasites, and while counts probably include a small number of immature sexual stages, we assume these to be negligible as before [6].

Using linear regression as described by [6], the time-varying growth *P*_*e*, *t*_ can be estimated for each cycle of proliferation within a host. We calculate effective propagation for each individual mouse (unlike in [6], which calculates an average across mice) by solving Eq 6 for Pe,t. Effective propagation numbers describe invasion success per infected red blood cell, which encompasses the number of progeny parasites released as well as their chances of contacting and invading susceptible red blood cells ([6], visual explanation in [33]). By incorporating red blood cell dynamics, effective propagation numbers yield better estimates of parasite proliferation than multiplication rates. Expanding on Eq 6, the gametocyte dynamics would be

$$G_{t+3}=c_{t}P_{e,t}I_{t}S_{t}$$ (7)

assuming that no gametocytes persisted from previous cycles and where *c*_*t*_ is again the transmission investment. Here the time lag is three proliferative cycles (each lasting one day) because the effective propagation number *P*_*e*, *t*_ describes the invasion success of parasites sampled at time *t*. Those parasites will give rise to another generation of infected red blood cells at time *t* + 1, of which some fraction *c*_*t*_ will have begun the process of sexual differentiation that will be complete by time *t* + 3. Since gametocytes are likely to carry over, we can add those terms:

$$G_{t+3}=c_{t}P_{e,t}I_{t}S_{t}+\epsilon G_{t+2}$$ (8)

with *ϵ* indicating the fraction of previously produced, mature gametocytes persisting to the current time point. While the number of mature gametocytes that persist is likely to vary through time, we assume that the distribution of gametocyte lifespans will remain constant. In particular, we assume that, upon attaining maturity, gametocyte lifespans follow an exponential distribution, as has been done in previous work to estimate gametocyte half-lives in *P. chabaudi*[20]. The fraction of mature gametocytes persisting to a subsequent time point can be estimated as a single constant, *ϵ*, which serves the dual purpose of describing gametocyte longevity (*ϵ* can be easily converted to a mean lifespan or half-life) and correcting conversion rates for gametocytes outside the cohort of interest. This method can be readily extended to *P. falciparum* and other species by modifying the time lags required for proliferation (Eq 6) and gametocyte development (Eq 8). No prior knowledge of gametocyte longevity is required, but if gametocyte mortality is expected to change through time—for example because of treatment with gametocytocidal drugs—then a single constant may not be sufficient to describe gametocyte survival and multiple *ϵ* values may be needed to describe different parts of the time series.

By analogy to susceptible reconstruction in epidemiology (e.g., [34]) we may recast Eq 8 as a cumulative recursion in terms of infected and susceptible cells:

$$G_{t}=\left(\sum_{j=t_{0}+3}^{t}\epsilon^{t-j}c_{j-3}P_{e,j-3}I_{j-3}S_{j-3}\right)+\epsilon^{t-t_{0}-2}G_{t_{0}+2},t_{0}\geq 1$$ (9)

where *t*_0_ is the first time point when effective propagation can be calculated, provided that gametocytes were censused at *t*_0_ + 2. In the simulated data, effective propagation can be calculated from the first day (thus, *t*_0_ = 1) and:

$$G_{t}=\left(\sum_{j=4}^{t}\epsilon^{t-j}c_{j-3}P_{e,j-3}I_{j-3}S_{j-3}\right)+\epsilon^{t-3}G_{3}$$ (10)

Mature gametocytes are first observed in the simulated data at the third time point, so *G*_3_ could be used as a starting point for subsequent time steps. However, in real data there is likely to be some error in the gametocyte counts *G*_3_ (or more generally, *G*_*t*_0_+2_) that would bias the fits to subsequent time points, so we fit those initial gametocyte counts as an additional parameter in the model.

Rather than fitting each *c*_*t*_ independently (which would be possible but extremely parameter-wasteful), we calculate the time-varying transmission investment as a smooth curve. Specifically, we use a sequence of splines of increasing complexity to describe the pattern of transmission investment and employ F-tests to determine when more complicated splines are justified by the data. To constrain transmission investment to biologically plausible proportions (i.e., between zero and one), we work with the complimentary log-log of the spline and consider five shapes: (1) constant; (2) linear; (3) parabolic; (4) cubic; (5) or a cubic spline with one interior knot. For time-varying transmission investment, any polynomial up to a particular order can be described by a linear combination of the spline basis functions of the same order (e.g., [35]), and the parameters specifying the linear combination can be found by optimizing the fit to observed gametocyte abundance. Splines of greater complexity should always be expected to fit better, and since the models are nested we can compare them by calculated the F-statistic, which follows an F distribution [36]:

$$F=\frac{\left(sse_{q}-sse_{p}\right)/\left(p-q\right)}{sse_{p}/\left(n-p\right)}∼F_{p-q,n-p}$$ (11)

where *n* is the number of observations used in the fitting, *p* and *q* are the number of parameters used in the more complicated and simpler models (respectively), and *sse* is corresponding the sum squared error of the best fit parameters for the two models. The F-test requires more observations of gametocyte abundance than parameters in the more complicated model (*n* > *p*). For example, a parabolic transmission investment strategy is specified by five parameters including the fraction of gametocytes persisting to the following day (*ϵ*) and the initial gametocyte abundance (*G*_*t*_0_+2_), so determining whether that pattern offers a significantly better fit to the data requires at least six days of gametocyte counts along with the corresponding red blood cell and parasite counts from three days prior. The **R** code for the calculations can be found in the Supporting Information (S2 Text, S1 Code).

**Fig 3:**
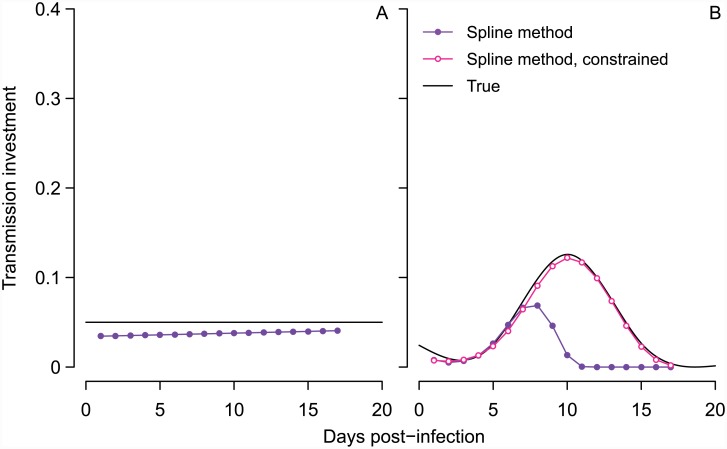
The spline method performs better than previous methods in capturing the true pattern of transmission investment. The true pattern is shown in black for constant (A) and variable (B) investment, while the spline estimate is indicated with closed purple circles for comparison to previous methods in Fig 2A and 2B. The gametocyte carryover (*ϵ*) corresponding to those estimated splines was 28% in (A), and 42% in (B), where the true value was 30%. In (B), constraining the gametocyte carryover to be less than 35% improved the spline fit (open pink circles), an improvement that is not possible for previous methods which do not account for carryover.

Encouragingly, we find that this elaboration of a time series SIR model [6] yields more reliable estimates of transmission investment (Fig 3). When we simulate data assuming constant transmission investment, the new method recapitulates the fixed transmission investment with relatively little bias (Fig 3A). Our new method modestly underestimates the true transmission investment because some gametocytes die by the time the infection is sampled. When we correct the gametocyte abundances for this mortality (i.e., by dividing by the proportion expected to survive from maturation at midnight to sampling), the estimated transmission investment is very close to the true value (S3 Fig). The estimated transmission investment tends to increase towards the end of infection because sampling occurs slightly earlier in the life cycle as the infection wears on (Fig 2, S2 Fig) due to the assumptions that asexual replication requires 24 hours from invasion to bursting and that subsequent merozoite invasion is rapid but not instantaneous. Thus invasion occurs slightly later in each successive cycle, resulting in fewer gametocytes lost by the time the population is sampled. Since this greater number of gametocytes cannot be accounted for in the effective propagation number, the spline method increases the estimated transmission investment to achieve a good fit to observed gametocyte abundance. This error is small and likely to be be negligible in reality, assuming that dynamics remain synchronized over the sampling period.

We apply the fitting algorithm to data simulated with time-varying transmission investment and find that the estimated curve reflects key features of the true curve (Fig 3B), but overestimates the gametocyte carryover (*ϵ*) and hence the impact of early transmission investment decisions on subsequent gametocyte dynamics. Carryover was initially allowed to vary between zero and 100%, and when we refit the model constraining gametocyte carryover to be less than 35%, the spline matches the true pattern very closely (Fig 3B). Previous experiments with *P. chabaudi* have assumed that gametocyte lifespans are exponentially-distributed to arrive at a mean half-life of 14 hours, corresponding to 30% gametocyte carryover [20]. We therefore assumed 30% carryover to simulate time series, and so constraining the algorithm to choose carryover less than 35% improved the model fit. In reality, there is substantial variability around the mean gametocyte half-life, especially when male and female gametocytes are considered separately [20], and extrapolating from those confidence intervals suggests that carryover could range from four to 67%. Further characterization of the distribution of gametocyte lifespans—including testing whether exponential distributions are a good approximation—would greatly enhance our ability to infer transmission investment.

As with previous efforts to estimate transmission investment, we make specific assumptions about when sexual differentiation can first be detected. There is still uncertainty about when PCR methods can first detect gametocyte development (reviewed in [2]), and once that issue is resolved, it may be necessary to use different time lags than those specified in Eqs 7–9 (*τ* parameter in S2 Text, S1 Code). Our alternative approach is also subject to the same limitations that have always applied to estimated transmission investment: the inferred pattern will be biased whenever there is differential mortality of sexual and asexual stages. However, the effective propagation number, *P*_*e*_ is reduced when immunity is constraining asexual proliferation [6]. Accordingly, making use of simulated data from a model that incorporates a host immune response, we find that our approach is able to cope with immune-mediated clearance of asexual parasites (S4 Fig).

**Fig 4:**
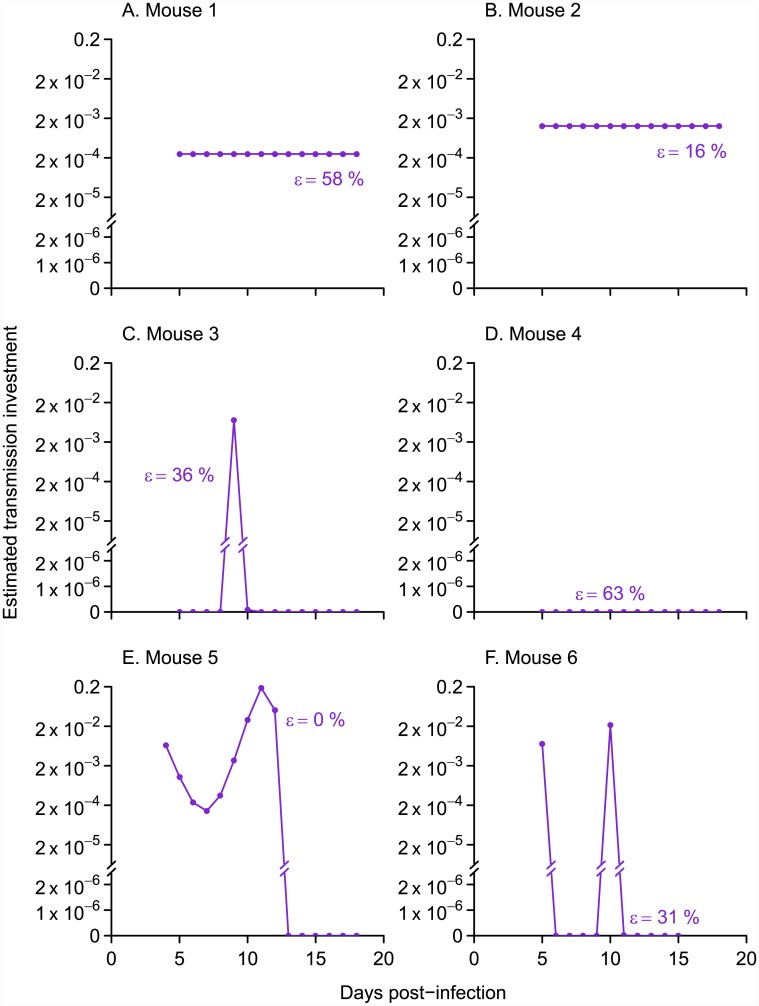
The spline method shows evidence for both constant and variable patterns of transmission investment in data from six mice. Points indicate the pattern of transmission investment associated with the best fit to logged gametocyte counts (S5 Fig). When the proportion gametocyte carryover (*ϵ*) was allowed to vary between zero and one (purple closed dots), three mice showed constant levels of transmission investment (A, B, and D) while the others showed variable patterns (C, E, and F). The corresponding levels of gametocyte carryover are given next to each curve, and all fall below the upper confidence limit reported previously (67%, equivalent to a 41-hour half life, [20]). The observed and predicted gametocyte counts are shown in S5 Fig.

In a first application of our approach, we analyze a published data set from mice infected with a *P. chabaudi* [37, 38], fitting the model to time series for individual mice. Our new method reveals highly variable patterns of transmission investment across mice (Fig 4). Three mice showed variable transmission investment over the time period sampled, while dynamics in the other three mice were adequately explained with a constant level of investment (observed and predicted gametocyte counts shown in S5 Fig). Of the mice with constant transmission investment, some but not all were predicted to have relatively high (though still plausible) levels of gametocyte carryover (58% and 63% in Fig 4A, and 4D versus 16% in B). Therefore the cases of constant transmission investment cannot be attributed solely to the model overestimating gametocyte carryover. Mouse 4 (Fig 4D) exhibited no evidence for any transmission investment over the period sampled. Specifically, for mouse 4, the model indicates that the most parsimonious explanation for the dynamics from day seven onwards—the time period for which transmission investment can be estimated—is that the observed gametocyte population was produced by parasite cohorts prior to day five and that some of those gametocytes persisted to subsequent days. No ongoing transmission investment is needed to explain the dwindling numbers of gametocytes observed (Fig 4D), analogous to the example presented in Fig 1C. The increase in gametocyte numbers from day six to day seven results from a combination of leftover gametocytes produced early in infection and newly-matured gametocytes produced by the day four cohort of parasites, but asexual counts are too low to yield reliable estimates of effective propagation. A key point is that the initial rate of increase in gametocytes in mouse 4 cannot be partitioned into transmission investment and carryover from previous cohorts because reliable estimates are lacking for the rate of proliferation in the progenitor cohort. The infection dynamics in this fourth mouse stand in contrast to the other mice in this treatment group (S6 Fig), including a notably greater level of anemia consistent with the inference that these parasites were allocating relatively more to proliferation rather than transmission.

Variable patterns of transmission investment have been reported previously, for models fit to time series of *P. falciparum* infections of human patients [29]. Yet the differences in transmission investment across mice are especially striking given that these infections represent genetically similar hosts inoculated with a uniform dose of the same parasite strain and housed in identical lab conditions. The variance across hosts is unlikely to be caused solely by stochastic differences in the initial inoculum size, which would have been accounted for in the calculation of effective propagation numbers. Previous work on *P. chabaudi* has shown greater variation in gametocyte counts across mice later in infection [39]. The increasing variance may be driven by differences in the immune response, which, when experimentally perturbed, can substantially alter gametocyte dynamics in mice [40]. Mice may differ in their adaptive immune responses, even to the same parasite strain, as has been shown in humans: naïve volunteers infected with a single strain of *P. falciparum* diverged in their immune responses, acquiring different sets of antibodies in response to the antigens expressed by parasites [41]. Thus, one possible explanation is that mice quickly diverge in their immune responses, despite being genetically homogenous, leading to large differences in transmission investment across mice.

### Concluding remarks

Our results suggest that estimating transmission investment is a more challenging problem than has previously been appreciated. We have focused on malaria infections in mice as a (comparatively) straightforward case study, because the system is amenable to experimental manipulation and the parasite life cycle has been extensively characterized. Even when synchronized cohorts of parasites can be identified, as with *P. chabaudi*, linking those cohorts to their subsequent transmission stage production is a nontrivial problem. Whenever transmission stages persist longer than a cycle of within-host proliferation—a complication likely to arise in diverse parasites—errors in transmission stage abundance are non-independent and more specialized statistical approaches are needed. The approach we develop here addresses this challenge and reveals intriguingly diverse patterns of transmission investment in real infections.

## Methods

All calculations were performed using **R** (R Project for Statistical Computing, http://r-project.org/). Unless otherwise noted, we used the model specifications of Greischar *et al*. [18]. The full details of the expanded age-structured model for gametocyte development is in the online Supporting Information (S1 Text), as is the annotated code for the new method of calculating transmission investment (S2 Text, S1 Code).

## Supporting Information

S1 Text Early markers for gametocyte development cannot remove bias in current methods. (PDF)

S2 Text Annotated code for the new method. (PDF)

S1 Code Executable R code for the new method. (R)

S1 Fig Early detection *per se* does not ensure that transmission investment will be correctly estimated. Here we assume that sexual differentiation can be detected as soon as a red blood cell is invaded, and the resulting abundance of infected red blood cells undergoing sexual differentiation (red) is compared with the total number of infected red blood cells (gray, A). Sampled time points are indicated by dots. The inferred transmission investment is shown below (B), taken as the fraction of the total number of infected red blood cells (excluding mature gametocytes) that are undergoing sexual differentiation (that is, *I_G_*(*t*)/(*I_G_*(*t*) + *I*(*t*))). The true transmission investment (5%) is shown as a dashed black line.
(EPS)

S2 Fig Early markers can resolve transmission investment under special conditions. The expression profile of the hypothetical marker during sexual development is shown in red (A, B). Parasites in the latter part of sexual development (i.e., not expressing the marker, gray) were excluded from calculations of transmission investment, along with mature gametocytes. Infection dynamics are shown on a log-scale (C, D), with marker-expressing parasites (early sexuals) shown in red and the total number of immature parasites (both early sexual and asexual forms) indicated by the black curves. “Sampling” of the simulated infection occurred at the same time each day (approximately six hours after peak-bursting and invasion) at the points indicated by dots. The resulting estimates for transmission investment are shown below in red (E, F), calculated as the proportion of marker-expressing parasites to total recently-invaded red blood cells (asexual or marker-expressing sexual). The actual level (5%) is indicated by a dashed line.
(EPS)

S3 Fig The estimated transmission investment is closer to the true value when we correct for gametocyte mortality. Specifically, we divide gametocyte abundance by *exp*(-*μ_g_* * 0.3), where *μ_g_* is the mortality rate for gametocytes, and 0.3 represents the time lag between synchronous bursting events and sampling. As before, transmission investment was estimated with splines, fitting the model to time series simulated with transmission investment pattern shown in black.
(EPS)

S4 Fig Estimated transmission investment in the presence of immunity against asexual parasites (purple dots), with the true value shown in black. As before, transmission investment curves of increasing complexity were fit to the simulated time series. Simulations assumed that immune clearance saturates as the number of asexual parasites increases (*a* = 150, *b* = 100).
(EPS)

S5 Fig Model-fitted gametocyte abundance (red curves) compared with observed gametocyte counts (black points). These fits correspond to the patterns of transmission investment shown in Fig 4.
(EPS)

S6 Fig Abundance of red blood cells (blue), infected red blood cells (red) and gametocytes (green) for the six mice used to estimate transmission investment. Mouse 4 showed slightly unusual dynamics, which are delineated with darker colors and broken lines. Data were taken from untreated infections with drug-resistant *P. chabaudi* parasites [37, 38].
(EPS)
