# Supplementary material for: Quantifying Transmission Investment in Malaria Parasites
Source: PLoS Comput Biol. 2016 Feb 18;12(2):e1004718. doi: 10.1371/journal.pcbi.1004718 (PMC4759450; doi:10.1371/journal.pcbi.1004718)
Supplement: S1 Text — (PDF) [file pcbi.1004718.s001.pdf]

Supporting information

Early markers cannot remove the bias

When immature sexual stages cannot be distinguished from asexual parasites, apparent transmission investment is likely to be biased [2], and early markers for sexual differentiation should therefore improve estimates of transmission investment. We first assume that some hypothetical gene is expressed as soon as a sexually-committed merozoite invades a red blood cell and that expression continues through sexual differentiation. We then imagine an expression-based genetic assay which can quantitatively estimate the number of parasites expressing that marker and which are therefore committed to developing into transmission stages. Unfortunately, our simulated data reveal that early detection by itself does not ensure accurate estimates of transmission investment (S1 Fig). While early markers avoid the problem of underestimating transmission investment due to mortality through development, the problem remains that developing gametocytes from different cohorts are lumped together.

Therefore a marker of early gametocyte differentiation may yield spurious oscillations in transmission investment if it continues to be expressed throughout development during the course of multiple rounds of asexual growth, as would be the case for the *Pfdgv1* gene in *P. falciparum*, for example [16]. An early molecular marker could still be useful in conjunction with a marker for late sexual differentiation, so that sexually differentiating parasites that emerged from previous rounds of bursting and invasion can be excluded from calculations of transmission investment. We expand the model—adding age structure to sexual differentiation—to identify an ideal expression pattern from which accurate transmission investment estimates could be obtained in *P. chabaudi* (details below). We assume that a molecular marker is expressed during early development, beginning immediately upon invasion, and that other data (a later marker or morphological differences) can be used to distinguish between parasites undergoing the early versus late stage of sexual differentiation. We consider two scenarios, in which the marker indicates sexual stages in the first 12 or first 24 hours of development. Since sampling is performed daily, this early marker means that sexual differentiation can be detected the same day as the current cohort of infected red blood cells were invaded by merozoites and no time lag needs to be taken into account.

When we consider a marker expressed during the first 12 hours of sexual differentiation, we see that the abundance of marked parasites undergoes large daily oscillations due to the high degree of synchrony and the brevity of the stage (S2 Fig, panels A, C), whereas when the marker is expressed for the first 24 hours,

there is less diurnal variation in abundance (S2 Fig, panels B, D). If the early marker is expressed for only 12 hours, then sampling at the same point in the life cycle becomes difficult following a very modest shift in timing over the simulation (S2 Fig, panels A, C). The time shift occurs because the model assumes that the asexual life cycle takes 24 hours (for asexual maturation) plus the time spent as a merozoite. The merozoite stage adds a very small amount of time to the life cycle, since merozoites are assumed to persist an average of only 30 minutes in the absence of red blood cells to invade; the length of the simulated merozoite stage is reduced further when red blood cells are abundant [18]. The precise timing and degree of synchronization of the parasite life cycle is not yet known for even the most well-studied species (reviewed in [17]), but it may be optimistic to assume that *P. chabaudi* does not deviate from its 24 hour cycle by more than half an hour. Still, even that small shift in timing over the simulated infection makes it impossible to measure abundance at the same point in the life cycle (S2 Fig, panel C), leading to increasing errors over time (S2 Fig, panel E). The difficulty in recovering the correct answer by existing methods is disappointing given that the simulations results represent an unrealistic extreme; in reality, there is likely to be variation in the length the parasite life cycle as well as observational errors.

Simulations suggest that transmission investment can be accurately estimated using a marker for the first 24 hours of gametocyte development (or the first 48 hours in *P. falciparum*), or in other words, a marker for sexual differentiation that exactly mirrors the timing of asexual development. Such a marker would work because it matches the mean duration and variation in duration experienced by parasites during asexual growth. If we assume that the first 24 hours of gametocyte development can be detected, and that this early developmental period is fixed in length, the fact that the life cycle is slightly longer than 24 hours alters the timing of early sexual stages and asexual stages in an identical manner, and so does not generate errors in transmission investment calculations. In contrast, using mature gametocytes to estimate transmission investment gives substantial errors, despite the fact that gametocytes survive nearly the length of the asexual life cycle on average (Fig. 2). Since the mean lifespan of the sexual stage is similar to that of asexual stages in both cases, the error is attributable to variation in the duration of the observed life stage. Although the mean lifespan is 20 hours, the constant mortality rate yields substantial variation about that mean, so that many gametocytes die as soon as they mature while a small fraction persist much longer than 20 hours. In contrast, asexual development is assumed to last exactly 24 hours, with no variation (i.e., a Dirac-Delta distribution of waiting times). Thus, while the mean lifespan of gametocytes and asexual stages is similar, the distribution of lifespans is markedly different, leading to

substantial error over the course of infection (Fig. 2A).

Thus current methods cannot be assumed to yield accurate estimates of transmission investment even in combination with early molecular markers, except under highly restrictive conditions. While early markers are available for the human malaria *P. falciparum* [16], it is not clear that those markers would reveal the underlying pattern of transmission investment, especially given that synchrony is lost over time *in vitro* [42] and at rates that vary across strains [43] and culture conditions [44]. Early markers have not yet been developed for *P. chabaudi*, and it is difficult to imagine a marker with the necessary expression profile. The key point is that early markers cannot by themselves address the major challenge of accurate inference: linking transmission-committed cells with their progenitor cohort.

Modeling age-structured gametocyte development

We model red blood cell (R) dynamics according to our previous model [18], with a constant merozoite (M) invasion rate p::

$$\frac{dR(t)}{dt} = \lambda\left(1 - \frac{R(t)}{K_{start}}\right) - \mu R(t) - pR(t)M(t). \tag{1}$$

Red blood cells are assumed to be depleted by infection and background mortality (μ) and replenished in a logistic fashion to maintain homeostasis, where λ is the maximum rate of erythropoiesis and $K_{start} = \lambda R^*/(\lambda - \mu R^*)$. After invasion by merozoites, a fraction $1 - c$ of the infected red blood cells will be committed to in-host replication (I class):

$$\frac{dI(t)}{dt} = (1-c)p(t)R(t)M(t) - \mu I(t) - \zeta(t) \tag{2}$$

where c is the transmission investment, i.e., the proportion of invaded red blood cells undergoing sexual differentiation. The null hypothesis is a constant level of transmission investment, unaffected by environmental factors, leaving c constant. While infected red blood cells are subject to background mortality, we assume no immune clearance, at least for the initial and simplest model validation. Infected red blood cells that survive through the life cycle are given by $\zeta(t)$:

$$\zeta(t) = \begin{cases} (1-c)pR(t-\alpha)M(t-\alpha)\exp(-\mu\alpha) & \text{if } t > \alpha \\ I_0 \text{Beta}(s_P, s_P)(t)\exp(-\mu t) & \text{if } t \le \alpha. \end{cases} \tag{3}$$

where α is the time required for asexual parasites to develop from invasion to bursting. We assume that within the initial inoculum parasite age follows a beta distribution, so that we can vary the overall degree of synchrony in development. A highly synchronized infection is simulated by setting $s_P = 100$. Each infected red blood cell that survives through the development period α days bursts to release β merozoites:

$$\frac{dM(t)}{dt} = \beta\zeta(t) - pR(t)M(t) - \mu_Z M(t). \tag{4}$$

Merozoites die at a high rate, μ_Z.

Our previously published model assumed a single developmental compartment for sexual differentiation I_G. To assess the consequences of detecting sexual differentiation early, mid-stage, or late, we split that compartment in three for the present analysis. Early sexual differentiation is given by

$$\frac{dI_E(t)}{dt} = cpR(t)M(t) - \mu I_E(t) - \zeta_E(t) \tag{5}$$

where

$$\zeta_E(t) = \begin{cases} cpR(t-\alpha_E)M(t-\alpha_E)\exp(-\mu\alpha_E) & \text{if } t > \alpha_E \\ 0 & \text{if } t \leq \alpha_E. \end{cases} \tag{6}$$

where α_E is the time required for early gametocyte development. Mid-stage sexual differentiation requires α_M days and is defined by

$$\frac{dI_M(t)}{dt} = \zeta_E(t) - \mu I_M(t) - \zeta_M(t). \tag{7}$$

where

$$\zeta_M(t) = \begin{cases} cpR(t-\alpha_E-\alpha_M)M(t-\alpha_E-\alpha_M)\exp(-\mu\alpha_E\alpha_M) & \text{if } t > \alpha_E+\alpha_M \\ 0 & \text{if } t \leq \alpha_E+\alpha_M. \end{cases} \tag{8}$$

Progression through the final stages of gametocyte differentiation is described by

$$\frac{dI_L(t)}{dt} = \zeta_M(t) - \mu I_L(t) - \zeta_L(t) \tag{9}$$

where

$$\zeta_L(t) = \begin{cases} cpR(t-\alpha_G)M(t-\alpha_G)\exp(-\mu\alpha_G) & \text{if } t > \alpha_G \\ 0 & \text{if } t \leq \alpha_G. \end{cases} \tag{10}$$

and $\alpha_G = \alpha_E + \alpha_M + \alpha_L$. Thus, α_L is the time required for late-stage gametocyte development and α_G is the total time required for gametocyte development after invasion of a red blood cell. After late-stage gametocyte differentiation, infected red blood cells become mature gametocytes (G):

$$\frac{dG(t)}{dt} = cp(t-\alpha_G)R(t-\alpha_G)M(t-\alpha_G)S_G - \mu_G G(t). \tag{11}$$

References

42. Trager W, Jensen JB. Human malaria parasites in continuous culture. Science. 1976 Aug;193(4254):673–675. Available from: http://www.ncbi.nlm.nih.gov/pubmed/16108535.

43. Reilly HB, Wang H, Steuter JA, Marx AM, Ferdig MT. Quantitative dissection of clone-specific growth rates in cultured malaria parasites. International Journal for Parasitology. 2007 Dec;37(14):1599–1607. Available from: http://www.pubmedcentral.nih.gov/articlerender.fcgi?artid=2268714&tool=pmcentrez&rendertype=abstract.

44. Allen RJW, Kirk K. *Plasmodium falciparum* culture: the benefits of shaking. Molecular and Biochemical Parasitology. 2010 Jan;169(1):63–65. Available from: http://www.ncbi.nlm.nih.gov/pubmed/19766147.
