# Supplementary material for: Quantifying Transmission Investment in Malaria Parasites
Source: PLoS Comput Biol. 2016 Feb 18;12(2):e1004718. doi: 10.1371/journal.pcbi.1004718 (PMC4759450; doi:10.1371/journal.pcbi.1004718)
Supplement: S2 Text — (PDF) [file pcbi.1004718.s002.pdf]

Supporting information: code for new approach

We first load the splines package and import the data as a csv file, obtained by downloading the data from Dryad [38] and saving it as a csv with no other alterations. Within **R**, the column names for the drug resistant strain (the strain we analyze here) are renamed so they can be quickly referenced later. Additionally, all parasite counts are given as number per μL, but uninfected counts are given in units of red blood cells $\times 10^6$, and we indicate that in the object `rbcUnit`. This algorithm can be used to analyze different data sets, provided that data is entered in the same format as the Dryad data set with one row per observation. For different data sets, the parts of the code that need to be altered at the column names below and the `rbcUnit` parameter. Specifically, the column names corresponding to red blood cell counts, asexual parasite counts, and gametocyte counts need to be renamed "RBC", "Asex" and "Gam", respectively. If the red blood cell counts are in different units, the `rbcUnit` parameter will need to be changed.

```
library(splines)
# import data:
allData = read.csv("SH1 dryad data file.csv", header=TRUE, sep = ',')
# focus on non-drug treated infections
nodrugs = allData[allData$Drugs=="nodrugs",]
# select resistant clone
clones = nodrugs[nodrugs$Clone=="R",]
# rename columns for drug resistant asexuals and gametocytes
  # for ease of referencing later
colnames(clones) <- c("Box","Mouse","Drugs","Clone","Day","Weight","RBC","Asex","S.asex","Gam",
"S.gam")
# set units for RBCs
  # in this data set, RBC numbers are given in numbers that need to be multiplied by 10^6
rbcUnit = 10^6
```

We first write a function to compare the nested models fitted to the data. This `getF` function returns a p-value from an F-test given sum squared errors for different model fits:

```
getF = function(n, nparms1,sse1,nparms2,sse2){
  # function to return p-value associated with F statistic
  # which follows an F distribution with df1 = p-q, df2 = n-p
  # first determine which model has more parameters/
  # smaller sum squared error:
  if(nparms1>nparms2 & sse2>sse1){
    sseOmega = sse1
    p = nparms1
    sseomega = sse2
    q = nparms2
  } # end test for model1 is more complex
  if(nparms2>nparms1 & sse1>sse2){
```

```
  sseOmega = sse2
  p = nparms2
  sseomega = sse1
  q = nparms1
} # end test for model2 is more complex
if(nparms2>nparms1 & sse2 > sse1){
  sseOmega = NA
  p = NA
  sseomega = NA
  q = NA
  print("Error: model fit is not a global optimum, refit with different starting parameters.")
} # if model2 has more parameters but doesn't fit better, that's an error
if(nparms1>nparms2 & sse1 > sse2){
  sseOmega = NA
  p = NA
  sseomega = NA
  q = NA
  print("Error: model fit is not a global optimum, refit with different starting parameters.")
} # if model2 has more parameters but doesn't fit better, that's an error
Fstatistic = ((sseomega-sseOmega)/(p-q))/(sseOmega/(n-p))
pVal = pf(q=Fstatistic, df1=(p-q), df2=(n-p), lower.tail=FALSE)
return(pVal)
} # end getF function
```

The function uses if/then statements to determine which model is more complicated (i.e., has more parameters), and then performs the F-test given in Eqn. 11 and returns the corresponding p-value. Models with more parameters should always give a smaller sum squared error, and if the more complicated model does not give smaller errors, it indicates that the global best fit has not been found. The `getF` function returns an error in those cases.

The following code fits splines of increasing complexity to time series data for a single mouse, here mouse 1. The `mouseID` is used to ensure that when output is written to files later on, each file name is unique and does not overwrite the output for other mice. The period required for gametocyte development is referred to as 'tau', and is set to two days [23], though it can be modified. The 'maxCarryover' refers to the maximum allowed ϵ value.

```
maxCarryover=1
i=1
mouse=clones[clones$Mouse==i,]
mouseID=i
tau=2
```

The method requires continuous, daily counts of red blood cells and parasite numbers, and the subsequent gametocyte counts, which can include missing values ('NA' in **R**). In this data set, mice were sampled intermittently later in the experiment. We use the *rle* function in **R** to find the longest run of

consecutive data.

```
# to get the longest continuous run of data:
 resLog=rle(diff(mouse$Day)==1)
 # returns lengths of runs for which the difference in plausible indices is 1
 runLength = max(resLog$lengths[which(resLog$values)]) # get the max length of
 # the consecutive run
 dummy=0
 j=1
 while(dummy==0 & j<length(mouse$Day)){
   startDay=mouse$Day[j]
   endDay=mouse$Day[(j+runLength)]
   if(startDay+runLength==endDay){
     startIndex=which(mouse$Day==startDay)
     dummy=1}
   j = j+1
 }
```

Once we have isolated the usable data, we estimate the effective propagation (here denoted x) using the methods described by [6]:

```
# regression approach
  # set up time lags for asexual dynamics
  times = mouse$Day[startIndex:(startIndex+runLength)]
  rbcUI = rbcUnit*mouse$RBC[startIndex:(startIndex+runLength)] # infected & uninfected RBCs
  para = mouse$Asex[startIndex:(startIndex+runLength)]
  rbc = rbcUI-para # uninfected RBCs ONLY
  gams = mouse$Gam[startIndex:(startIndex+runLength)]
  Itplus1 = para[2:length(para)]
  It = para[1:(length(para)-1)]
  St = rbc[1:(length(rbc)-1)]
  # Estimate effective propagation numbers
  x = rep(NA, length(It))
  for (p in c(1:length(It))){
    if(is.na(It[p])==FALSE && is.na(Itplus1[p])==FALSE){
      if(It[p]>0 & Itplus1[p]>0){
        fitA = lm(log(Itplus1[p])~offset(log(It[p])+log(St[p])))
        x[p] = exp(fitA$coef[[1]])}
    }
  } # end asex loop
  x[x*St>20]=NA
  length(x) <- length(times) # so that an NA is added for the last day,
  # when effective propagation could not be estimated
```

Some of the effective propagation numbers will be unrealistically large due to the difficulty of accurately quantifying small numbers of parasites. For example, in mouse 1, asexual parasite numbers appear to increase more than 100-fold from day 4 to day 5, despite the fact that maximum burst sizes are on the order of ten to 20 [45]. We can locate the faulty data by calculating the effective reproductive number (the effective propagation number multiplied by the number of susceptible red blood cells), which represents

the number of emerging parasites that successfully invade red blood cells and should be less than the maximum burst size, here set to 20. Any implausible effective propagation numbers are recoded as missing values. We can then isolate the part of the data set with plausible effective propagation numbers:

```
subtimes = times[is.na(x)==FALSE]
 subx = x[is.na(x)==FALSE]
 subrbc = rbc[is.na(x)==FALSE]
 subpara = para[is.na(x)==FALSE]
 subgams = gams[is.na(x)==FALSE]
 resPe=rle(diff(subtimes)==1)
 # returns lengths of runs for which the difference in plausible indices is 1
 runPeLength = max(resPe$lengths[which(resPe$values)]) # get the max length of
 # the longest run
 dummyPe=0
 jPe=1
 while(dummyPe==0 & jPe<length(subtimes)){
   startPeDay=subtimes[jPe]
   endPeDay=subtimes[(jPe+runPeLength)]
   if(startPeDay+runPeLength==endPeDay){
     startPeIndex=which(subtimes==startPeDay)
     dummyPe=1}

   jPe = jPe+1 }
 tvals = subtimes[(startPeIndex):(startPeIndex+runPeLength)]
 xt=subx[(startPeIndex):(startPeIndex+runPeLength)]
 rbcToFit = subrbc[(startPeIndex):(startPeIndex+runPeLength)]
 paraToFit = subpara[(startPeIndex):(startPeIndex+runPeLength)]
 gamToFit = subgams[(startPeIndex):(startPeIndex+runPeLength)]
```

An effective propagation number calculated for time t can be used to predict gametocyte counts at $t + \tau + 1$. If transmission investment occurs on day t, invasion of committed parasites and initial gametocyte development would have occurred when parasite populations are sampled on day $t + 1$, and gametocyte development would have been completed when samples are taken on day $t + 3$. There may be additional gametocyte counts that can be included in the analysis even if effective propagation could not be estimated for those days, because those gametocytes would have been the result of effective propagation occurring earlier. This code adds those extra gametocyte counts to the data to be fitted:

```
for (extraDays in c(1:(tau+1))){ # add one to tau because commitment
 # occurs one day prior to gametocyte development
 extrat = startPeDay+runPeLength+extraDays
 # test that the extra day was included in the data set
 if(extrat%in%mouse$Day){
   gamIndex=which(mouse$Day==extrat)
   # test that gam count is included in the data set,
   if(is.na(mouse$Gam[gamIndex])==FALSE){
     gamToFit=append(gamToFit,mouse$Gam[gamIndex])}
 } # end conditional
```

```
  } # end for loop
  length(rbcToFit) <- length(gamToFit)
  length(paraToFit) <- length(gamToFit)
  dataToFit = cbind(rbcToFit,paraToFit,gamToFit)
  numObs = length(which(is.na(gamToFit)==FALSE))-tau # the first tau observations are unusable
  convTime = seq(tvals[1],by=1,length=(length(gamToFit)-tau-1)) # days corresponding to the
  # transmission investment to be estimated
  gamCountTime = seq(tvals[1]+tau,by=1,length=(length(gamToFit)-tau))
  # (because there is no corresponding effective propagation number,
  # and the (tau+1) entry of the gamToFit vector serves as the initial gametocyte count)
```

We also count the number of gametocyte counts that can be used to fit the models because it could constrain the complexity of candidate splines. The first few gametocyte counts (i.e., the first τ days) cannot be used because there are no corresponding data to estimate effective propagation.

Now we can estimate transmission investment by fitting splines of increasing complexity. We first set the guess for the gametocyte mortality rate (`mug`) to an estimate from the literature, with the range of subsequent guesses falling within the confidence intervals reported [20], and then determine the candidate splines that are appropriate for the time series available. The starting guesses for the initial gametocyte count follow at gamma distribution as described previously for *P. chabaudi* gametocytes [37]. For all of the mice analyzed here, the time series were long enough to fit a cubic spline with an interior knot (the most complicated spline considered).

```
# best guess for gametocyte mortality rate
  mug = (log(2)/14)*24
  candidateSplines = c(0,1,2,3,5) # degrees of freedom needed for candidate splines
  nParmList = c(1:5)+2 # associated number of parameters
  # print notification if there are too few observations to fit even constant conversion:
  if(numObs<3){print("Error: too few observations to fit simplest model.")}
  # no point in fitting splines that are too complicated to be compared with F-tests:
  if(min(nParmList)<=numObs){
    degrees = candidateSplines[nParmList<numObs]
    if(min(nParmList)==numObs){degrees = min(candidateSplines)}
    bestFit = as.data.frame(matrix(NA, ncol = (length(degrees)+5), nrow = length(degrees)))
    colnames(bestFit) <- c(paste("p",c(1:(length(degrees)+2))),"numParms",
                           "numObs","sse")
    for (val in c(1:length(degrees))){
      dg=degrees[val]
      print(dg)
      if(dg<4){
        if(dg==0){sVals = matrix(0.5,ncol=1,nrow = length(convTime))}
        if(dg>0){sVals = bs(convTime,degree=dg,intercept=TRUE)}}
      if(dg>=4){sVals = bs(convTime,df=dg,intercept=TRUE)}
      # define objective function after creating sVals spline object
      gobs <- function(parms,data){
        cparms = parms[1:(length(parms)-2)]
        cVal = exp(-exp(sVals%*%cparms))
```

```
  epsilon = maxCarryover*exp(-exp(parms[(length(parms)-1)]))
  G3est = exp(parms[(length(parms))])
  Stminus3 = data[,1]
  Itminus3 = data[,2]
  Gt = data[(tau+1):length(Stminus3),3]
  GtPred = rep(NA, length(Gt))
  GtPred[1] = G3est
  for (Gindex in c(2:length(GtPred))){
    j=c(1:(Gindex-1))
    GtPred[Gindex]=sum((epsilon^((Gindex-1)-j))*(cVal[j])*xt[j]*
                         Itminus3[j]*Stminus3[j])+(epsilon^(Gindex-1))*G3est
  }
  GtPred[GtPred>1e20]=1e20
  sse <- sum((log(GtPred+1)-log(Gt+1))^2,na.rm=TRUE)
} # end gobs fx
# loop to estimate conversion rates by finding coefficients
# of spline basis functions
tries = 1000 # tries to fit lowest number of parameter values
mugHi = (log(2)/5)*24
mugLo = (log(2)/41)*24 # confidence intervals from Reece et al. 2003
minEps = exp(-mugHi) # minimum allowable epsilon
maxEps = exp(-mugLo) # maximum allowable epsilon
parms0 = c(runif(ncol(sVals),min=-45,max=85),log(-log(runif(1, min=minEps, max = maxEps))),
 log(1+rgamma(1,shape = 1/0.0582,scale = 0.0582*gamToFit[(tau+1)])))
parmTries = matrix(NA, nrow = tries, ncol = length(parms0)+1)
for (take in c(1:tries)){
  dummy=1
  while(dummy!=0){
    convFit <- optim(parms0, gobs, data=dataToFit, control=c(maxit=5000))
    dummy=convFit$convergence
    parms0 = c(runif(ncol(sVals),min=-45,max=85),log(-log(runif(1, min=minEps, max = maxEps))),
     log(1+rgamma(1,shape = 1/0.0582,scale = 0.0582*gamToFit[(tau+1)])))
    sseVal = gobs(convFit$par, data=dataToFit)
    print(c(i,take,convFit$convergence,parms0))
  } # end while loop to get convergence
  parmTries[take,] = c(convFit$par,sseVal)
} # end loop to get multiple convergent tries with different
# starting parameters
# set bar for sse
if(degrees[val]==min(degrees)){minSSE = min(parmTries[,(length(parms0)+1)])}
if(degrees[val]>min(degrees)){
  while(min(parmTries[,(length(parms0)+1)])>minSSE){
    take=take+1
    parms0 = c(runif(ncol(sVals),min=-45,max=85),log(-log(runif(1, min=minEps, max = maxEps))),
     log(1+rgamma(1,shape = 1/0.0582,scale = 0.0582*gamToFit[(tau+1)])))
    convFit <- optim(parms0, gobs, data=dataToFit, control=c(maxit=5000))
    print(c(i,take,convFit$convergence,parms0))
    if(convFit$convergence==0){
      sseVal = gobs(convFit$par, data=dataToFit)
      newTry = c(convFit$par,sseVal)
      parmTries = rbind(parmTries,newTry)}
  } # end while loop
}
bestrow = parmTries[which.min(parmTries[,(length(parms0)+1)]),1:length(parms0)]
```

```
      parms = bestrow
      bestFit[val,1:length(convFit$par)] = parms
      numParms = length(convFit$par)
      bestFit$numParms[val] = numParms
      cparms = parms[1:(length(parms)-2)]
      cVal = exp(-exp(sVals%*%cparms))
      filelab = paste("ConversionMouse", i, "Degree", dg, ".txt", sep = '')
      write.table(cVal,filelab)
      epsilon = maxCarryover*exp(-exp(parms[(length(parms)-1)]))
      G3est = exp(parms[(length(parms))])
      Stminus3 = dataToFit[,1]
      Itminus3 = dataToFit[,2]
      Gt = dataToFit[(tau+1):length(Stminus3),3]
      bestFit$numObs[val] = numObs

      GtPred = rep(NA, length(Gt))
      GtPred[1] = G3est
      for (Gindex in c(2:length(GtPred))){
        j=c(1:(Gindex-1))
        GtPred[Gindex]=sum((epsilon^((Gindex-1)-j))*(cVal[j])*xt[j]*
                          Itminus3[j]*Stminus3[j])+(epsilon^(Gindex-1))*G3est
      }
      GtPred[GtPred>1e20]=1e20 # end predicting G by gobs algorithm

      sse <- sum((log(GtPred+1)-log(Gt+1))^2,na.rm=TRUE)
      minSSE=sse
      bestFit$sse[val] = sse
      res = as.data.frame(cbind(Gt,GtPred))
      write.table(res, paste("ConversionMouse", i, "Degree", dg, "Pred.txt", sep = ''))
      # plot fit
      # get index of any NAs in gam counts
      gamNA = which(is.na(Gt))
      par(mfrow = c(1,1), bty = "n", mar = c(5,5,3,1))
      plot(gamCountTime, log(Gt+1)-log(GtPred+1), pch = 16, col = "black",
           xlab = "t", ylab = "Residual gametocyte abundance",
           main = paste("degree = ", dg, sep = ""))
      if(any(is.na(Gt))){
        points(gamCountTime[gamNA],rep(0,length(gamNA)),pch = 4, col = "red")
      }
    } # end degree loop
    # write results table
    resLab = paste("SplineFitsMouse", i, ".txt", sep = '')
    write.table(bestFit,resLab)
  } # end conditional regarding number of data points
```

This code loops through increasingly complex splines to find the best parameters, and we set up a table (`bestFit`) to hold the summary values we obtain from those fittings, including up to seven parameter values associated with the splines, proportion of gametocytes persisting to subsequent samples (ϵ), and initial number of gametocytes observed, the number of parameters in each model, the number of observations in the data, and the sum squared error (`sse`). We then construct the spline basis functions for

a given degree (the matrix `sVals`), and define the objective function for *optim* to evaluate the sum-squared error (`gobs`). The key part of the code is the `gobs` function, which predicts the gametocyte count according to Eqn. 9 given parameters for the spline, `epsilon` and the initial number of gametocytes. The sum squared error is then calculated from the logged gametocyte abundances, since gametocyte counts can span many orders of magnitude. We add one to observed and predicted gametocyte counts in case either is zero. Within the objective function, we cap the maximum number of predicted gametocytes at 1×10^{20} in case a starting value yields an infinite number of gametocytes.

There is no guarantee that the optimization algorithm will return a global optimum, so to improve the odds we try many randomly chosen starting values, at least 1000 runs with convergence (`convFit$convergence`$= 0$). Since we cycle through nested models, we can detect that the algorithm has failed to locate a global optimum when a model with more parameters does not improve the fit. After 1000 attempts at fitting a constant investment model, we save the sum squared error associated with the best fit as `minSSE`. We add a *while* loop to continue trying different starting values until the algorithm locates a fit with a sum squared error less than `minSSE`, a plausible global best fit. We then set `minSSE` equal to this new, smaller sum squared error and proceed to fit more complicated splines.

For each fitted spline, the code takes the best parameters found, reconstructs the spline (which would not have been saved in the successive optimizations) and exports it as a text file under a file name indicating the mouse and degree of the spline (for example, "ConversionMouse1Degree3.txt"). The code recalculates the predicted gametocyte abundances and exports them along with the observed values so that the fits can be examined later (e.g., "ConversionMouse1Degree3Pred.txt"). A plot of the residuals for each best fitting spline is generated. Finally, when the five candidate splines have been fit for a given mouse, the `bestFit` table is exported ("SplineFitsMouse1.txt").

Afterwards, the `bestFit` tables can be used to determine whether more complicated models are justified by the data. We use forward model selection, meaning that we take the simplest model (constant investment) and compare it to each more complicated model to determine if the fit is significantly better ($p < 0.05$), using the `getF` function defined earlier. If the fit is better, we then select that model and compare more complicated models to determine whether more parameters are justified. Once the appropriate model is selected, the code reads in the transmission investment, as well as the observed and predicted gametocyte abundances as "estConv1" (for mouse 1) and exports those numbers for use later on.

The best fitting pattern of transmission investment can then be plotted. This code is available as an executable **R** file (S1 Code).

```
 # run F-test
  res = read.table(paste("SplineFitsMouse",i,".txt",sep=''))
  m=1
  k=2
  pVal=1
  forwardseln = 1
  while(k<=length(res[,1]) & pVal>0.05){
    pVal = getF(res$numObs[1],res$numParms[m],res$sse[m],res$numParms[k],res$sse[k])
    print(c(m,k,pVal))
    if(pVal<0.05){forwardseln=k; m=k; pVal=1}
    k=k+1
  }
  bestPar = res[forwardseln,1:(res$numParms[forwardseln])]
  bestEpsFull = maxCarryover*exp(-exp(bestPar[(length(bestPar)-1)]))
  bestEps = c(bestEpsFull[[1]])
  conv = read.table(paste("ConversionMouse",i,"Degree",degrees[forwardseln],".txt",sep=''))
  bestConv = conv[,1]
  gamEst = read.table(paste("ConversionMouse",i,"Degree",degrees[forwardseln],"Pred.txt",sep=''))
  length(convTime) <- length(gamCountTime)
  length(bestConv) <- length(gamCountTime)
  length(bestEps) <- length(gamCountTime)
  res.output = as.data.frame(cbind(convTime,bestConv,gamCountTime,gamEst$Gt,gamEst$GtPred,bestEps))
  colnames(res.output) <- c("Days","Conversion","GamDays","Gt","GtPred","epsilon")
  assign(paste("estConv",i,sep=''),res.output)
  write.table(res.output,paste("estConv",i,".txt",sep=''))

plot(estConv1$Days,estConv1$Conversion, type = "o", col = "purple3",pch=16)
```

References

45. Mideo N, Savill NJ, Chadwick W, Schneider P, Read AF, Day T, et al. Causes of variation in malaria infection dynamics: insights from theory and data. The American Naturalist. 2011 Dec;178(6):E174–E188. Available from: http://www.ncbi.nlm.nih.gov/pubmed/22089879.
